# Depression and Vegetarians: Association between Dietary Vitamin B6, B12 and Folate Intake and Global and Subcortical Brain Volumes

**DOI:** 10.3390/nu13061790

**Published:** 2021-05-24

**Authors:** Samuel Berkins, Helgi Birgir Schiöth, Gull Rukh

**Affiliations:** 1 Department of Neuroscience, Functional Pharmacology, Uppsala University, BMC, Box 593, 751 24 Uppsala, Sweden; samuel.berkins@gmail.com (S.B.); Helgi.Schioth@neuro.uu.se (H.B.S.); 2 Institute for Translational Medicine and Biotechnology, Sechenov First Moscow State Medical University, 119991 Moscow, Russia

**Keywords:** vegetarians, vitamin B6, vitamin B12, depression, brain structure

## Abstract

Deficiency of vitamin B6 and vitamin B12, mostly in vegetarians, is found to be associated with depression and adverse neurological function. We investigated whether vitamin B6, B12, and folate have an effect on brain structure, especially among depressed people who follow a specific diet. The study sample comprised 9426 participants from the UK Biobank cohort with a mean age of 62.4 years. A generalized linear model controlling for age, sex, body mass index, ethnicity, town send deprivation index, educational qualification, smoking, and alcohol intake was used to test the association between study groups and structural brain volumes. Depression was more prevalent, and intake of vitamin B6 and B12 was lower among vegetarians, while non-vegetarians had a lower intake of folate. Overall, no significant association was observed between vitamin B6, B12, and folate intakes and both global and subcortical brain volumes among participants with depression. However, vitamin B12 intake was positively associated with right pallidum among non-depressed participants, and a significant interaction between vitamin B12 intake and depression status on the right pallidum was observed. Also, a significant interaction between folate intake and depression status on grey matter (GM) volume and left thalamus was observed. Upon diet stratification, folate intake is associated with total brain volume and GM volume among vegetarians with depression. Furthermore, no significant associations were observed for subcortical regions. Our findings suggest that dietary intake of vitamin B6 and B12 might have an effect on brain structure. Vegetarians, particularly those who suffer from depression may benefit from supplementing their diets with vitamins B6, B12, and folate to ensure brain health. Further studies, especially with a larger sample size and longitudinal design, are needed to confirm these findings.

## 1. Introduction

Depression is a heterogeneous psychiatric disorder affecting millions of people across the globe. It is speculated to be the leading cause of disease burden by 2030 [1], affecting both men (22%) and women (28%) over 65 years of age [2]. It can occur in individuals of any age from childhood to late life, causing tremendous economic burden and can be fatal if left untreated. Nevertheless, the aetiology and neurobiological underpinnings of depression are not clearly understood.

There is growing evidence that specific dietary patterns may be a key factor for the onset of depression [3]. Studies have reported a higher rate of depression among vegetarians compared to non-vegetarians [4,5,6]. For example, Hibbeln et.al. reported that vegetarians are more depressed due to nutritional deficiencies such as cobalamin or iron [5] and are more neurotic compared to non-vegetarians [6]. However, during the last few years, the attraction towards vegan or vegetarianism has risen due to associated health benefits and moral values towards society [7]. Moreover, a recent survey found an increase of 2.6% in vegans in just one decade [8]. This trend is alarming as it raises a potential concern of increasing the risk of vitamins B6 and B12 deficiency among vegetarians since the main dietary source of these nutrients is meat and seafood.

Previously, increased deficiency of vitamins B6 and B12 has been reported among vegetarians [9,10]. Some studies have also linked vitamin B6 and B12 deficiency with adverse neurological function [11,12]. Moreover, it has been shown that reduced levels of vitamin B12 and folate causes increased total homocysteine (tHcy) levels, leading to a variety of cardiovascular diseases and cerebrovascular conditions among the aging population [13,14,15]. Furthermore, elevated levels of tHcy adversely affect mental health, e.g., direct neurotoxic effect or the impact of cerebrovascular pathology [14,16]. It is because vitamin B12 and folate are the main components involved in the remethylation of homocysteine to methionine, and inadequate intake of either vitamin B12 or folate may lead to an escalated concentration of tHcy [17]. In contrast, supplementation of vitamin B6 and B12 was associated with reduced depression events [18].

Neuroimaging studies on the aging population have shown an inverse relationship between vitamin B12 and total brain volume (TBV) loss over 60 years of age [19]. A longitudinal study comprising 146 older adults (>65 years) found that the Mediterranean diet was significantly associated with preserved white matter (WM) volume, along with the reduced occurrence of depression [20]. Moreover, a voxel-based morphometric study (based on 32 community-dwelling adults) on vitamin B6 and B12 has shown that adults with higher vitamin B6 intake had increased grey matter (GM) volume along the medial wall, anterior cingulate cortex, medial parietal cortex, middle temporal gyrus, and superior frontal gyrus, and adults with higher vitamin B12 intake had greater volume in the left and right superior parietal sulcus [21].

Briefly, the current literature provided evidence for the increased deficiency of vitamin B6 and B12 among vegetarians [22,23] and established the increased prevalence of depression among vegetarians compared to non-vegetarians [4,6]. However, most of these studies are based on a small sample size and are limited to the occurrence of depression rather than its impact on brain structure. Therefore, further studies with a larger sample size are needed to better understand the impact of specific food habits on brain structure and mental health, especially among people with depression. The UK Biobank (UKB) cohort is an emerging resource of ~500,000 participants with rich phenotyping data including; physical and mental health measures, information on diet and lifestyle factors, as well as brain and body imaging [24].

The current study aimed to investigate the association between dietary vitamin B6, B12 and folate intake on global and subcortical brain volumes after stratifying for depression status using data from the UKB. We further aimed to investigate how belonging to a specific diet group (vegetarians and non-vegetarians) affects this association.

## 2. Materials and Methods

### 2.1. Study Sample

**Figure 1:**
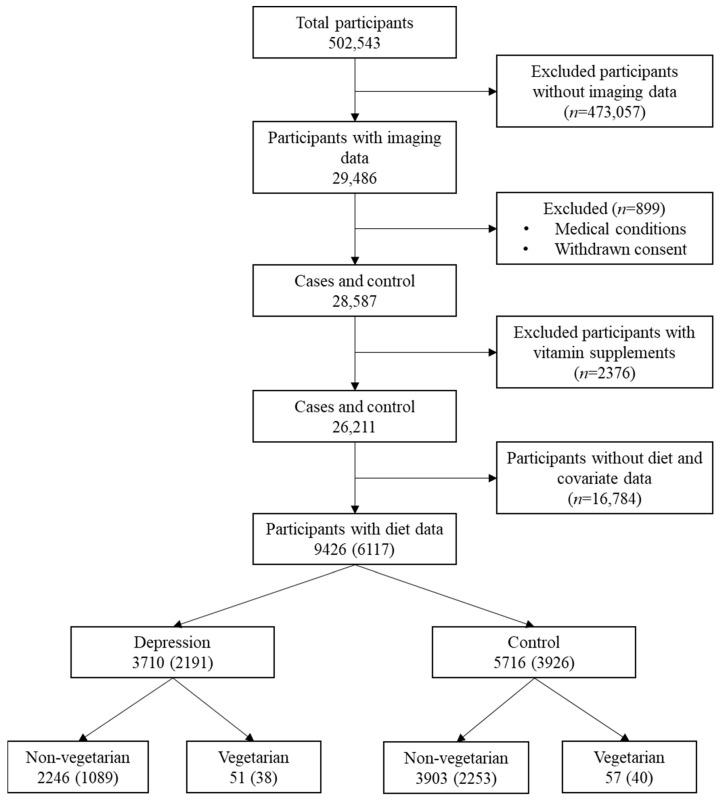
Study population. The values in parenthesis represent the sample for sensitivity analyses in which participants undergoing major dietary changes in the past were further excluded. The information regarding dietary status was not available for everyone in the study population.

UKB is a population-based cohort consisting of 502,543 participants recruited at 22 different centres across the United Kingdom between 2006 and 2010. The UKB study was approved by the North West Multi-Centre Research Ethics Committee and by the Regional Ethics Committee of Uppsala, Sweden, and all participants provided written informed consent [24]. Starting in 2014, after the initial recruitment, some of the participants were re-invited to participate in the brain, heart, and body imaging. In the present study, we used brain Magnetic Resonance Imaging (MRI) data acquired between 2014 and 2019 that was made available to us under the UKB application: 23482. At the time of the acquisition, information regarding processed MRI data was available for 29,486 participants, and a cross-sectional design was adopted. We excluded all the participants (*n* = 899) who have withdrawn consent or having any neurological conditions except depression, body mass index (BMI) < 18.5 as well as participants with certain medical conditions that might potentially lead to the deficiency of vitamin B6, B12, and folate (See Supplementary Information). Moreover, all the participants taking vitamin supplements were excluded to study the exclusive effect of diet (*n* = 2376). Following all the exclusion criteria, the final participants count included in the study with available dietary data was 9426 (Figure 1).

### 2.2. Study Measures

#### 2.2.1. Imaging Data

In the UKB, imaging data were collected by employing Siemens skyra 3T running VD13A SP4 and using Siemens 32-channel RF head coil. The T1 weighted structural imaging phenotype was acquired through straight sagittal orientation with a resolution of 1 × 1 × 1 mm^3^ and field-of-view (FoV) of 208 × 256 × 256 matrix. All T1-weighted structural images were processed through a fully automated pipeline based on FMRIB Software Library (FSL) software [25]. A detailed description of the image quality control, image analysis pipeline, and brain image processing can be viewed at https://biobank.ctsu.ox.ac.uk/crystal/crystal/docs/brain_mri.pdf (accessed on 1 April 2020) [26]. In brief, the T1 images were automatically accessed for wrong dimensions, corruption, missing or probable brain artifacts through a supervised machine learning model, and the images were classified as usable and non-usable. If the images were classified as non-usable, they were not processed further. The images that were categorized as usable were defaced, and the FoV was cut down to reduce the no-brain tissue using the brain extraction tool [27] and the FMRIB’s Linear Image Registration Tool [28]. Next, the nonlinear image is warped to the MNI152 template, and the warped image is segmented into the cerebrospinal fluid, grey matter, and white matter using FMRIB’s Automated Segmentation Tool (FAST) [29]. The brain tissue is then normalized to head size and can be accessed as Imaging Derived Phenotypes (IDPs) from the UKB database. Furthermore, the subcortical brain volumes are modelled using FMRIB’s Integrated Registration and Segmentation Tool [30] and available as IDPs in the UKB database. By use of several tools, structural numerical volume estimates for TBV (UKB field ID: 25009), GM (UKB Field ID: 25005) and WM (UKB Field ID: 25007), as well as subcortical volumes (UKB category ID: 1102; total 14 regions) were derived. All these IDPs are accessible upon request from the UKB database.

#### 2.2.2. Diet Data

In UKB, participants were presented with a web-based 24-h dietary assessment during April 2009, and the dietary assessment was mailed once every 3–4 months between 2011 and 2012. In the dietary assessment, participants were asked to estimate their total nutrients intake yesterday through food and beverages, excluding any supplements. The web-based dietary assessment questionnaire can be accessed through the UKB website (http://www.ukbiobank.ac.uk/key-documents/ (accessed on 1 April 2020)). Diet information that was mainly focused in our study included intakes of vitamin B6 (UKB Field ID: 100012), vitamin B12 (UKB Field ID: 100013), and folate (UKB Field ID: 100014) and was extracted from the web-based dietary assessment.

Information regarding dietary variation (UKB Field ID: 1548) was extracted from the question “Does your diet vary much from week to week” with five possible options: ‘Never/rarely’, ‘Sometimes’, ‘Often’, ‘Do not know’ and ‘prefer not to answer’. And the dietary variation variable was dichotomized into ‘Never/rarely’ and vary (categories ‘sometimes’ and ‘often’ were merged). For information regarding the dietary change (UKB Field ID: 1538), participants were asked the question, “Have you made any major changes to your diet in the last 5 years?” with four possible options: ‘No’. ‘Yes, because of illness’, ‘Yes, because of other reasons’ and ‘Prefer not to answer’. And the dietary change variable was dichotomized into no and yes (categories ‘Yes, because of illness’ and ‘Yes, because of other reasons’ were merged). All the participants registering their responses as ‘Do not know’ or ‘Prefer not to answer’ to both of the above questions were considered missing. Thus, all the participants who reported dietary change were excluded, and dietary variation was used as a covariate in all the analyses.

#### 2.2.3. Depression and Dietary Groups

We choose the broad category of depression using both hospital record data as well as self-reported touchscreen response to either question: “Have you ever seen a general practitioner (GP) for nerves, anxiety, tension or depression?” (UKB Field ID: 2090) and “Have you ever seen a psychiatrist for nerves, anxiety, tension or depression?” (UKB Field ID 2010). Cases were determined, if the response was “Yes” to either one of the questions, or if there was a primary or secondary diagnosis of a depressive mood disorder from hospital admission records (UKB Field ID: 41202 and 41204; ICD 10 codes: F32–F34, F38–F39) [31].

For the classification of study participants into dietary groups (vegetarians and non-vegetarians), there are no direct dietary questions in the UKB. However, data is available regarding the frequency of consumption of major food groups. Thus, by combining information based on relevant questions from the touchscreen dietary questionnaire, participants were assigned to one of the two groups. Briefly, participants were identified as vegetarians if they did not report consuming red meat (the sum of questions related to the intake of beef, lamb, and pork), poultry, any types of fish, or processed meat. Non-vegetarians were identified as those who reported eating red meat, processed meat, poultry, or any types of fish (see Supplementary Materials for a detailed description on the classification of vegetarians and non-vegetarians) [32].

### 2.3. Study Covariates

Other covariates that were taken into account were ethnicity, Townsend Deprivation Index (TDI), BMI, alcohol intake, smoking, education, and physical activity. The association of covariates with TBV is shown in Table S1. The metabolic equivalent of the Task (MET) score was extracted from the touchscreen-based questions, which have been described in detail previously [33]. Educational qualification was recoded from qualifications (UKB Field ID: 6138) into a binary variable after categorizing participants with and without a university degree.

### 2.4. Statistical Analyses

All analyses were conducted using a statistical package for social sciences SPSS, version 26. From all the continuous variables. extreme outliers were removed (with a z-score exceeding ±3.29 for the mean intake of vitamin B6 and B12, folate, and brain volumes). Chi-square test was used to test the relationship between depression status and diet groups. Association between intake of vitamin B6, B12 and folate, and structural brain volumes (GM, WM, TBV, and subcortical volumes) was studied using a general linear model with brain volumes as outcomes and dietary intakes as determinants of main interest after stratifying for depression status and dietary groups. The interaction effect was also investigated between depression/dietary groups and vitamin B6, B12, and folate intake on GM, WM, and TBV by introducing a multiplicative term in the model without stratification.

We applied three different models: Model 1 was adjusted for age, sex, BMI, and ethnicity, Model 2 was adjusted for all the covariates in model 1 plus total energy intake, dietary variation, and educational qualification, and Model 3 (fully adjusted model) was adjusted for model 2 covariates plus socioeconomic status (TDI) and lifestyle factors (smoking, alcohol, and physical activity (MET score). Analyses with subcortical volumes were additionally adjusted for TBV. Sensitivity analyses were carried out after excluding participants, who have undergone major dietary changes in the last five years. The results obtained through all the models were almost similar; thus, sensitivity results of the fully adjusted model are presented throughout the manuscript. A two-sided *p*-value of <0.05 was considered significant in all the analyses concerning global brain volumes (GM and WM, and TBV). After correction for multiple testing (Bonferroni correction), a *p*-value of <0.0036 (0.05/14) was considered significant in analyses with subcortical brain volumes.

## 3. Results

Of the 9426 participants included in the present study, 3309 participants reported undergoing major dietary changes in the past five years. The characteristics of the study participants with no major dietary changes (*n* = 6117) are presented in Table 1. The mean age of participants was 62.38 (standard deviation (SD) ± 7.44) years, and 51.1% were women. Participants reported a mean intake of 2.18 ± 0.63 mg, 6.3 ± 3.51 µg, and 303.15 ± 94.97 µg of vitamin B6, B12, and folate respectively, which was estimated through food and beverages consumption after excluding supplements.

### 3.1. Mean Intakes of Vitamin B6, B12 and Folate Intake Stratified by Depression Status and Diet Groups

**Figure 2:**
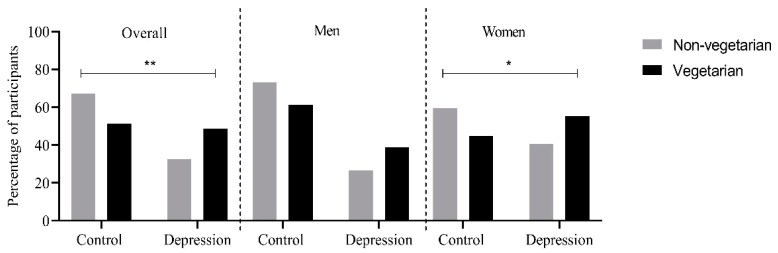
Percentage of vegetarians and non-vegetarians suffering from depression in the overall population and after stratifying for sex. * represent *p* < 0.05 ** represent *p* < 0.01.

Overall, depression was more prevalent in vegetarians (48.7%) compared to non-vegetarians (32.6%), and a significant difference in depression status was observed between diet groups (*p* = 0.003). The same trend was observed in men and women separately, and the prevalence of depression was higher among women compared to men in both diet groups. Also, among women, depression was more prevalent among vegetarians (*p* = 0.042) (Figure 2). The mean intakes of vitamin B6, B12, and folate stratified by depression status (cases and control) and dietary groups (vegetarians and non-vegetarians) are shown in Table 2. Overall, a significantly higher intake of vitamin B6 ((mean ± SD): 2.19 ± 0.62 mg; *p* = 1.36 × 10^−7^), vitamin B12 (6.37 ± 3.46 µg; 1.2 × 10^−21^) and a significantly lower intake of folate (301.76 ± 93.65 µg; 0.002) was observed among non-vegetarians. However, in the case of depression, no significant difference was observed in the mean intakes of vitamin B6, B12, and folate (Table 2).

### 3.2. Association of Nutrient Intake, Depression and Diet Status with Brain Volumes

We first investigated the main effects of nutrient intake (vitamin B6, B12, and folate), depression, and diet status (vegetarians and non-vegetarians), on global and subcortical volumes. In the unadjusted analyses, nutrient intakes and depression status were significantly associated with GM, WM, and several subcortical volumes (Tables S2 and S3). However, after adjustment with study covariates, significant associations were observed only for folate intake (β ± SE: −13.57 ± 5.42; *p*-value: 0.01) and depression status (β ± SE: 2096.40 ± 944.44; *p*-value: 0.03) with GM (Table S2).

### 3.3. Association of Vitamin B6, B12 and Folate Intake with Brain Volumes Based on Depression Status

#### 3.3.1. Global Volumes

**Figure 3:**
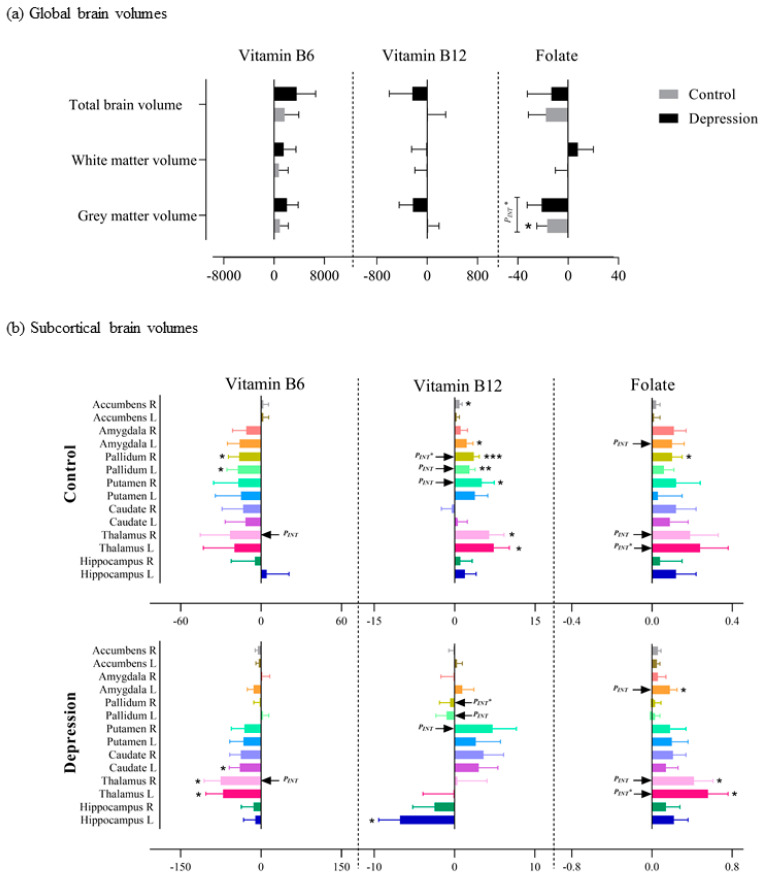
Association of vitamin B6, B12 and folate intake with (**a**) global brain volumes and (**b**) subcortical brain volumes among depression cases and controls. P_INT*_ signifies the interaction between vitamin intake and depression status on brain volumes after Bonferroni correction (i.e., for (**a**) *p* < 0.05 and for (**b**) *p* < 0.0036). *p* < 0.05 = *, *p* < 0.01 = ** and *p* < 0.001 = ***.

We observed a significant association between folate intake and GM among controls (β ± SE: −16.61 ± 8.32; *p*-value: 0.045) and a significant interaction effect between folate intake and depression status on GM (*P_INT_*: 0.02). No significant associations were observed between folate intake and WM or TBV and between vitamin B6 and B12 intake and global brain volumes in either depression cases or controls. Moreover, no other significant interaction effect was observed (Table S4 and Figure 3a).

#### 3.3.2. Subcortical Volumes

For the subcortical brain volumes, nominal significant associations were observed between vitamin B12 intake and left pallidum (β ± SE: 2.80 ± 1.01; *p*-value: 0.005), right putamen (5.04 ± 2.32; 0.03), right accumbens (0.93 ± 0.47; 0.048), left amygdala (2.29 ± 1.14; 0.045) and bilateral thalamus (left: 7.31 ± 2.90; 0.01 and right: 6.43 ± 2.65; 0.01) in the control group. In depression cases, we observed a nominal significance association between vitamin B12 intake and left hippocampus (−6.78 ± 2.65, 0.01) (Table S5 and Figure 3b). Vitamin B6 nominally associated with bilateral pallidum (left: −17.34 ± 8.08; 0.03 and right: −16.35 ± 7.96; 0.04) in the control group and with bilateral thalamus (left: −70.83 ± 31.80; 0.03 and right: −75.25 ± 30.57; 0.01) and left caudate (−39.51 ± 19.65; 0.04) in the depression group (Table S5 and Figure 3b). Nominal significant associations were observed between folate intake and right pallidum (0.10 ± 0.05; 0.048) in the control group and between folate intake and bilateral thalamus (left: 0.56 ± 0.20; 0.005 and right: 0.42 ± 0.19; 0.03) and left amygdala (0.18 ± 0.07; 0.02) in the depression group. However, after correction for multiple testing, only right pallidum (3.61 ± 1.00; 2.0 × 10^−4^) was significantly associated with vitamin B12 intake, and a significant interaction was observed between vitamin B12 intake and depression status on the right pallidum (0.001) (Table S5 and Figure 3b). In addition, a significant interaction was observed between folate intake and depression status on the left thalamus (0.0032) (Table S5 and Figure 3b).

### 3.4. Association of Vitamin B6, B12 and Folate Intake with Brain Volumes after Stratifying for Depression Status and Diet Groups

#### 3.4.1. Global Volumes

**Figure 4:**
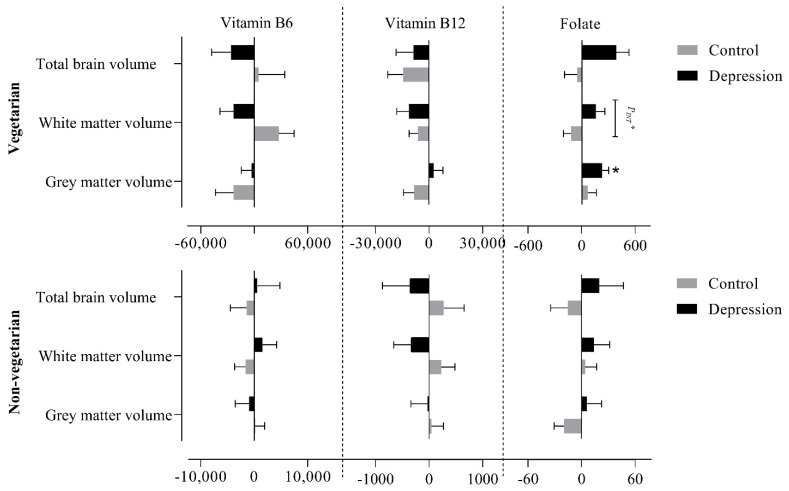
Association between vitamin B6, B12 and folate intake with global brain volumes (grey matter, white matter and total brain volume) after stratifying for depression status and diet groups (vegetarians and non-vegetarian). *P_INT_* signifies the interaction between vitamin intake and depression status on brain volumes (P_INT_^*^ = *p* < 0.05; *p* < 0.05 = *).

We further stratified the data based upon the dietary status of the participants, i.e., vegetarians and non-vegetarians. In vegetarians with depression, a significant positive association of folate intake with GM (227.80 ± 76.10; 0.01) and with TBV (388.45 ± 143.52; 0.01) was observed (Table S6 and Figure 4). In addition, a significant interaction was observed between folate intake and depression status on WM (0.02) among vegetarians. Among non-vegetarians, no significant association was observed between nutrient intake and global brain volumes in either group (depression and control), and no significant interaction was observed between vitamin B6, B12, and folate intakes, and depression status on global brain volumes (Table S6 and Figure 4).

#### 3.4.2. Subcortical Volumes

**Figure 5:**
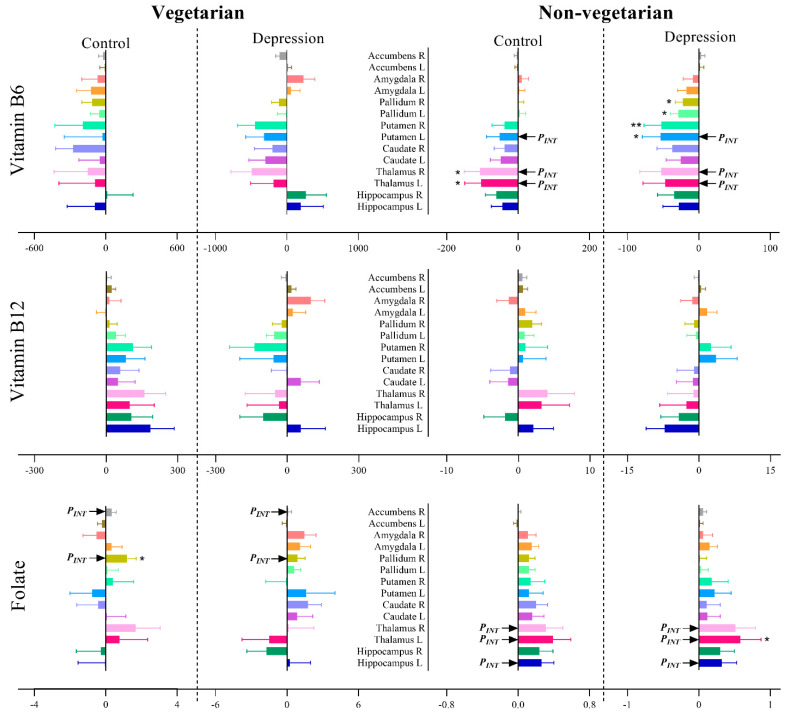
Association of vitamin B6, B12 and folate intake with subcortical brain volume after stratification for dietary status and depression. *P_INT_* signifies the interaction between vitamin intake and depression status on subcortical brain volume (*P_INT_* = *p* value < 0.05 but >0.0036), *p* < 0.05 = * and *p* < 0.01 = **.

Among vegetarians, folate intake showed nominal significance association with right pallidum (1.20 ± 0.50; 0.03) among the controls. Among non-vegetarians, vitamin B6 intake was nominally associated with bilateral thalamus (left: −103.80 ± 45.46; 0.02 and right: −106.55 ± 43.95; 0.02) in the control group and with bilateral putamen (left: −53.49 ± 25.43; 0.04 and right: −51.97 ± 24.67; 0.04) and bilateral pallidum (left: −28.85 ± 10.87; 0.008 and right: −22.29 ± 10.45; 0.03) in the depression group. Furthermore, the left thalamus was nominally associated with folate intake (0.58 ± 0.29, 0.046) among non-vegetarians with depression (Table S7 and Figure 5). In addition, nominal significant interactions were observed for vitamin B6 and folate intake with depression status on bilateral thalamus among non-vegetarian (*P_INT_* ≤ 0.04 for all). No associations were observed between vitamin B12 intake and subcortical volumes among both vegetarians and non-vegetarians (Table S7). After correction for multiple testing, no significant association or interaction was observed between nutrient intakes and subcortical brain volumes in any of the studied groups (Table S7 and Figure 5).

## 4. Discussion

In this study, the association between dietary vitamin B6, B12, and folate intake on global and subcortical brain volumes after stratifying for dietary groups (vegetarians and non-vegetarians) and/or depression status was investigated. In our study population from the UK Biobank cohort, depression was more prevalent, and dietary intakes of vitamin B6 and vitamin B12 were lower among vegetarians compared to non-vegetarians. Overall, no significant association was observed between vitamin B6, B12, and folate intakes and both global and subcortical brain volumes among participants with depression. However, folate intake negatively associated with GM and vitamin B12 intake positively associated with right pallidum among non-depressed participants. Upon diet stratification, folate intake is positively associated with GM and TBV among vegetarians with depression. For subcortical brain volumes, no significant associations were observed for nutrient intakes in both depression and diet stratified analyses. Moreover, significant interactions were observed between vitamin B12 intake and depression status on the right pallidum and between folate intake and depression status on GM volume and left thalamus.

In agreement with the existing literature [4,5,6], depression was more prevalent among vegetarians compared to non-vegetarians in the present study. It has been shown that the deficiency of vitamin B12 leads to decreased neurotrophins level, resulting in increased oxidative stress, suggesting depression-associated risk in vegetarians [34]. This pattern suggests that belonging to a specific diet group or, in other words, specific dietary components may have an effect on depression status. The prevalence of depression status among women was 15 to 20% higher than men once the population was stratified for sex. These findings are consistent with research linking increased depression status among women [35], with two plausible explanations. Firstly, hyperactivity of the hypothalamic-pituitary-adrenal (HPA) axis has been shown to be associated with depression [36], and women are more susceptible to have stress-induced dysregulation of the HPA axis than men, which in turn increase the probability of stress-induced depression among women [37]. Secondly, androgen receptors in the hippocampal neurons of men may grant protection against depression [38].

Numerous studies have shown the importance of vitamin B6, B12, and folate on mental health [18,19]. However, to the best of the author’s knowledge, to this day, no such studies have been carried out to investigate the association between vitamins B6, B12, and folate intake and brain structures specifically based on depression status and diet classification. In our study population, we observed a significant difference in mean vitamin intake between vegetarians and non-vegetarians, which could explain a higher proportion of depressed participants among vegetarians. Public health of England recommends on average males and females within the age range of 19–74 years require 1.4 mg of vitamin B6, 1.5 µg of vitamin B12, and 200 µg of folate per day [39], which was derived from dietary reference values for Food Energy and Nutrients from United Kingdom (1991). Surprisingly, all participants in both diet groups and depression status met or surpassed the recommended daily intake of the vitamins by public health of England, yet the prevalence of depression status was more pronounced among vegetarians. Moreover, the National Institute of Health recommends a little higher intakes of these nutrients as on average males and females within the age group of 51–70 years require 1.7 mg of vitamin B6, 2.45 µg of vitamin B12, and 400 µg of folate per day [40]. Interestingly, all participants in both diet groups and depression status met or surpassed the recommended daily intake of the vitamin b6 and b12 but not folate by the National Institute of Health. This raises the potential concern whether the current recommended daily dietary vitamin intake by several public health governances is optimal or needs reconsideration.

In contrast with the previously published findings, we did not observe any association between dietary intake of vitamin B6 and B12 with GM, WM, or TBV. However, there was a significant association between folate intake and GM among non-depressed participants, and a significant interaction was also observed between folate intake and depression status on GM. One potential explanation for this discrepancy is the decreased bioavailability and poor absorption of vitamin B12 from food sources, especially in the aging population [41]. This might result in reduced vitamin absorption even though the daily recommended vitamin intake was met, leading to increased tHcy plausibly. Moreover, an earlier study has shown that increased concentration of folate serum combined with reduced vitamin B12 is associated with increased brain atrophy [42] and a higher risk of cognitive impairment [43,44,45].

In our study, we observed depressed participants following a vegetarian diet displaying positive association of folate intake with GM and TBV. However, these findings should be interpreted with caution as in our study, the vegetarian group having depression is very small that warrants further investigation. The observed association could be caused due to the role played by folate in the remethylation of homocysteine to methionine, in turn decreasing the tHcy level [17,46], and studies have revealed the direct neurotoxic effect caused due to elevated tHcy [14,16].

We observed a significant positive association between vitamin B12 intake and right pallidum among participants without depression. This finding is in line with the existing literature, as pallidum is a part of the limbic loop in basal ganglia, contributing to the reward system, regulation of motivational salience, behaviours, and emotions [47]. We also observed significant interaction between vitamin B12 and depression status on the right pallidum. A clinical report showed that a drug-addicted patient with lesions on ventral pallidum showed depressed mood and recovered from drug cravings and remained away from recreational drugs once the lesion was removed [48]. Furthermore, the ventral pallidum receives dopaminergic inputs from the ventral tegmental area and also receives GABAergic inputs from nucleus accumbens acting as a relay between nucleus accumbens and medial dorsal nucleus through which rewarding effects are mediated [49]. Certainly, our findings support the association observed in the above clinical reports, further insisting on the importance of dietary vitamin B12 intake for mental health.

The main strengths of this study are the relatively large dataset by UK Biobank and the vast resources such as well captured imaging and diet data, availability of several factors which could confound the association (such as dietary variation, dietary change, alcohol consumption, etc.). The reader should bear in mind that the associations and interaction effects observed in our study are solely based upon dietary vitamin intake and not due to vitamin supplement intake. In addition, the analyses were performed using various models to clearly analyse the effect of specific confounding variables on the observed associations. Excluding most of the possible biasing elements that could have a positive or inverse effect on the study, in turn, strengthened our findings on the association between dietary nutrient intake and brain volumes. Furthermore, association analyses were carried out after excluding participants who had undergone major dietary change during the last 5 years, to decrease type I error. Furthermore, the participants were classified into specific diet patterns through an actual dietary questionnaire which is more accurate rather than depending on basic questions (e.g., ‘are you following any specific diet pattern like vegan or vegetarian diet, etc.’) which may overestimate the associations [50,51].

However, some important limitations must be considered when interpreting the study findings. The vitamin intake data we used was not objectively measured but was self-reported by participants, which might have introduced reporting bias [52,53]. Even though the dietary questions were collected at four time points with a time gap of minimum of four months, the MRI scan was carried out only once at a different time point limiting the possibility of drawing causality. Specifically, as it is difficult to interpret whether dietary pattern preceded or succeeded the depression status. Moreover, several investigations have been carried out to show the efficiency and reliability of the food frequency questionnaire (FFQ) [50,51], but numerous discrepancies have been observed with the FFQ, and a frequently observed phenomenon is the underreporting of food intake [54]. Therefore, blood measures of dietary intake are a more reliable and unbiased nutritional validation method, as their measurement error is not corresponding with the test method [55]. Usage of blood measures would have strengthened our findings, but the blood levels of vitamin B6, B12, and folate are not currently available in the UK Biobank resource. Furthermore, studies have reported a positive correlation between nutrients estimated from dietary intakes with those of blood levels. Jacques et al. compared the intakes of 12 micronutrients obtained through semi-quantitative FFQ with corresponding biochemical measures and found a correlation coefficient of >0.30 for vitamin B12, folate, and some other micronutrients [56]. The reader should bear in mind that the study only shed light on the association between vitamin B6, B12, and folate, and brain volume, and interaction between depression status and vitamin B6, B12, and folate on brain volume focusing on the broad depression phenotype and not revealing any pathogenesis or aetiology of depression. However, given the small sub-sample size among diet groups (especially vegetarians), it cannot be determined whether the specific dietary pattern is detrimental to mental health. Taking into account the large unbalance between sample size for vegetarians and non-vegetarians, sex-stratified analyses were not performed, which could help us to further understand the role of studied intakes on neurological health based on physiological differences among men and women. As discussed above, future work should focus on utilizing more objective methods of nutrition assessment, such as obtaining blood measurements rather than using the subjective dietary intake data. In addition, a follow-up MRI scan would help to better understand the long-term impact of diet. Last but not least, future studies should focus on strict diagnosis of depression based on formal structured diagnostic assessment, instead of using a broader depression phenotype as used in this study, in order to eliminate any potential bias associated with the ascertainment of depression cases.

## 5. Conclusions

Our study is the first of its type carried out to understand the association between dietary intake of vitamin B6, B12, and folate with depression on global and subcortical regions stratified for depression status and dietary groups. We observed some positive associations between vitamin B6, B12, and folate intakes and brain volumes. The findings of our cross-sectional study provide a rationale for hypothesizing that adopting a specific dietary pattern may be associated with a nutritional deficiency that may have an impact on brain health. Vegetarians, particularly those who suffer from depression, may benefit from supplementing their diets with vitamins B6, B12, and folate to prevent loss of brain volume and to ensure better mental health. Further studies, especially with a larger sample size and longitudinal design, are needed to confirm these findings.

## Figures and Tables

**Table 1:** Characteristics of the Study Participants.

Characteristics (*n*)	Total (6117)	Depression (2191)		Control (3926)	
		Non-Vegetarian (1089)	Vegetarian (38)	Non-Vegetarian (2253)	Vegetarian (40)
Age (Year)	62.38 ± 7.44	61.62 ± 7.46	60.84 ± 7.09	62.57 ± 7.34	58.75 ± 6.71
Sex					
Male	3124 (48.9%)	514 (47.15%)	12 (31.58%)	1409 (62.51%)	19 (47.50%)
Female	2993 (51.1%)	575 (52.85%)	26 (68.42%)	845 (37.49%)	21 (52.50%)
Body Mass Index	26.03 ± 4.05	26.51 ± 4.27	25.66 ± 4.89	26.04 ± 3.91	25.55 ± 3.90
Smoking					
Never	4044 (66.1%)	689 (63.24%)	21 (55.26%)	1535 (68.10%)	32 (80.00%)
Previous	1878 (30.7%)	359 (33.00%)	15 (39.47%)	643 (28.53%)	7 (17.50%)
Current	195 (3.2%)	41 (3.77%)	2 (5.26%)	76 (3.37%)	1 (2.50%)
Alcohol					
Daily	1163 (19.0%)	225 (20.68%)	8 (21.05%)	448 (19.88%)	5 (12.50%)
3–4 times a week	1931 (31.6%)	314 (28.86%)	6 (15.79%)	758 (33.63%)	7 (17.50%)
1–2 times a week	1631 (26.7%)	268 (24.63%)	12 (31.58%)	636 (28.22%)	14 (35.00%)
1–3 times a month	634 (10.4%)	119 (10.94%)	3 (7.89%)	205 (9.09%)	6 (15.00%)
Special occasions	480 (7.8%)	109 (10.02%)	4 (10.53%)	135 (5.99%)	5 (12.50%)
Never	278 (4.5%)	54 (4.87%)	5 (13.16%)	72 (3.19%)	3 (7.50%)
Townsend derivative index	−2.26 ± 2.44	−2.14 ± 2.46	−1.72 ± 3.01	−2.39 ± 2.37	−1.62 ± 2.70
Dietary variation					
No	2353 (38.5%)	391 (35.85%)	15 (39.47%)	891 (39.55%)	15 (37.50%)
Yes	3763 (61.5%)	698 (64.15%)	23 (60.53%)	1362 (60.45%)	25 (62.50%)
Vitamin B6 intake	2.18 ± 0.63	2.17 ± 0.62	1.87 ± 0.75	2.21 ± 0.63	1.77 ± 0.50
Vitamin B12 intake	6.3 ± 3.51	6.33 ± 3.51	2.44 ± 1.14	6.41 ± 3.44	2.75 ± 1.60
Folate intake	303.15 ± 94.97	298.34 ± 93.97	340.89 ± 131.67	303.42 ± 93.47	327.90 ± 98.34

Values are expressed as mean ± SD for continuous variable and as N (percentage) for categorical variables.

**Table 2:** Mean dietary intake of vitamin B6, B12 and folate in the UK Biobank participants with imaging and diet data.

	Vitamin B6 (mg)			Vitamin B12 (µg)			Folate (µg)		
	Overall	Men	Women	Overall	Men	Women	Overall	Men	Women
Diet classification									
Sample	3420	1953	1467	3420	1953	1467	3420	1953	1467
Non-vegetarian	2.19 ± 0.62	2.26 ± 0.64	2.10 ± 0.59	6.37 ± 3.46	6.55 ± 3.55	6.14 ± 3.32	301.76 ± 93.65	311.73 ± 94.99	288.28 ± 90.09
Vegetarian	1.81 ± 0.63	1.91 ± 0.56	1.75 ± 0.67	2.60 ± 1.39	2.55 ± 1.45	2.62 ± 1.37	334.23 ± 115.20	351.61 ± 111.1	322.76 ± 117.57
*p*-Value	**1.36 × 10^−7^**	**0.002**	**6.0 × 10^−5^**	**1.2 × 10^−21^**	**4.8 × 10^−10^**	**8.0 × 10^−13^**	**0.002**	**0.021**	**0.01**
Depression status									
Sample	6117	3124	2993	6117	3124	2993	6117	3124	2993
Controls	2.19 ± 0.63	2.26 ± 0.64	2.09 ± 0.6	6.34 ± 3.49	6.49 ± 3.55	6.14 ± 3.38	304.37 ± 95.01	312.80 ± 95.3	293.23 ± 93.5
Cases	2.16 ± 0.62	2.27 ± 0.65	2.09 ± 0.59	6.21 ± 3.55	6.43 ± 3.74	6.06 ± 3.43	300.96 ± 94.86	317.67 ± 98.37	289.55 ± 90.68
*p*-Value	0.15	0.61	0.87	0.18	0.68	0.55	0.17	0.2	0.28

Values are expressed as Mean ± SD. Values were calculated based on the self-reporting of vitamin intake yesterday over four different time points. Association between vitamin intakes with diet classification or depression status was tested with analysis of variance. Values in bold represent statistically significant findings.

## Data Availability

The data supporting these results can be obtained from the UK Biobank cohort upon request.

## Supplementary Materials

The following are available online at https://www.mdpi.com/article/10.3390/nu13061790/s1, Table S1: Association between covariates and total brain volume, Table S2: Association of dietary intakes, depression and diet status with global brain volumes, Table S3: Association of dietary intakes, depression and diet status with subcortical brain volumes, Table S4: Association of vitamin B6, B12 and folate intake with global brain volumes based on depression status, Table S5: Association of vitamin b6, b12 and folate intake with subcortical brain volumes based on depression status, Table S6: Association of vitamin b6, b12 and folate intake with global brain volumes after stratifying for depression status and diet groups, Table S7: Association of vitamin b6, b12 and folate intake with subcortical brain volumes after stratifying for depression status and diet groups.
