# Supplementary material for: Depression and Vegetarians: Association between Dietary Vitamin B6, B12 and Folate Intake and Global and Subcortical Brain Volumes
Source: Nutrients. 2021 May 24;13(6):1790. doi: 10.3390/nu13061790 (PMC8225207; doi:10.3390/nu13061790)
Supplement: Supplementary file 1 [file nutrients-13-01790-s001.zip › nutrients-1177879-supplementary.pdf]

SUPPLEMENTARY INFORMATION

Depression and vegetarians: Association between dietary vita-min B6, B12 and folate intake and global and subcortical brain volumes

Samuel Berkins, Helgi Birgir Schiöth and Gull Rukh

Phenotype definition

1. Depression
    - ❖ Cases
        - From touchscreen questionnaire at recruitment
            - "Have you ever seen a GP/psychiatrist for nerves, anxiety, tension or depression?" (UKB FID 2090 and 2100) – **Yes**
        - From Hospital records from UK bodies
            - Any primary or secondary diagnoses of ICD-10 codes for mood disorders
            - F32 – Single Episode Depression
            - F33 – Recurrent Depression
            - F34 – Persistent mood disorder
            - F38 – Other mood disorders
            - F39 – Unspecified mood disorders
    - ❖ Control
        - From touchscreen questionnaire at recruitment
            - "Have you ever seen a GP/psychiatrist for nerves, anxiety, tension or depression?" (UKB FID 2090 and 2100) – **No**
2. Dietary Groups
    - From dietary touchscreen questionnaire
        - A dichotomous variable was created for each of the following variables from UKB by combining answers (i.e. 'Less than once a week', 'once a week', '2-4 times a week', '5-6 times a week', 'once ore more daily') to Yes and answer ('Never') to No and excluded participants responding 'Do not Know' and 'Prefer not to answer'.
            - ▪ 1369 – Beef intake
            - ▪ 1379 – Lamb/mutton intake
            - ▪ 1389 – Pork intake
            - ▪ 1359 – Poultry intake
            - ▪ 1329 – Oily fish intake
            - ▪ 1339 – Non-oily fish intake

        - 1349 – Processed meat intake.
- ❖ Non-vegetarian
    - The participants were categorised as non-vegetarian even if one of the responses to the dichotomous variable was **Yes**.
- ❖ Vegetarians
    - The participants were categorised as vegetarian if all the responses to the dichotomous variables were NO.

Exclusion Criteria

1. For both depression cases and controls
- o Bipolar (ICD codes F30, F31 or non-cancer illness code 1291)
- o Multiple personality disorder (ICD code F44.8)
- o Schizophrenia / psychosis (ICD codes F2*, or non-cancer illness code 1289)
- o Vitamin deficiency (ICD codes D51)
- o Disorder with vitamin malabsorption (ICD codes K29, D51 or non-cancer illness code 1462, 1461, 1224, 1552, 1373 and 1381)
- o Treatment/medication codes for antipsychotics and medication interacting with vitamin absorption (Field 20003):
    - Cimetidine 1140865426
    - Famotidine 1140865608
    - Ranitidine 1140879406
    - Metformin 1140884600
    - Rabeprazole 1141168584
    - Omeprazole 1140865634
    - Esomeprazole 1141177526
    - Lansoprazole 1140864752
    - Pantoprazole 1140929012
    - abilify 5mg tablet 1141202024
    - amisulpride 1141153490
    - aripiprazole 1141195974
    - benperidol 1140867078

- camcolit 250 tablet 1140867494
- carbagen sr 200mg m/r tablet 1141171566
- carbamazepine 2038459704
- carbamazepine product 1140872064
- chlorpromazine 1140879658
- clopixol 2mg tablet 1140867342
- clozapine 1140867420
- clozaril 25mg tablet 1140882320
- convulex 150mg e/c capsule 1140872216
- cpz - chlorpromazine 1140910358
- denzapine 25mg tablet 1141200458
- depakote 250mg e/c tablet 1141172838
- dolmatil 200mg tablet 1140867306
- dozic 1mg/ml oral liquid 1140867180
- epilim 100mg crushable tablet 1140872200
- fentazin 2mg tablet 1140867210
- fluphenazine decanoate 1140867398
- fluphenazine 1140882098
- haldol 5mg tablet 1140867184
- haloperidol 1140867168
- largactil 10mg tablet 1140863416
- levomepromazine 1140909802
- liskonum 450mg m/r tablet 1140867498
- lithium product 1140867490
- lithonate 400mg m/r tablet 1140910976
- methotrimeprazine 1140867118
- modecate 12.5mg/0.5ml oily injection 1140867456
- olanzapine 1140928916
- orlept 200mg e/c tablet 1140872268
- pericyazine 1140867134
- perphenazine 1140867208

        - pimozide 1140867218
        - piportil depot 50mg/1ml oily injection 1140867572
        - pipothiazine 1140879674
        - pipotiazine 1140909804
        - priadel 200mg m/r tablet 1140867504
        - prochlorperazine 1140868170
        - promazine 1140879746
        - quetiapine 1141152848
        - risperdal 0.5mg tablet 1141177762
        - risperidone 1140867444
        - serenace 500micrograms capsule 1140867092
        - seroquel 25mg tablet 1141152860
        - sodium valproate 1140872198
        - stelazine 1mg tablet 1140867244
        - stemetil 5mg tablet 1140868172
        - sulpiride 1140867304
        - tegretol 100mg tablet 1140872072
        - thioridazine 1140879750
        - trifluoperazine 1140868120
        - valproic acid 1140872214
        - zaponex 25mg tablet 1141201792
        - zuclopenthixol 1140882100
        - zyprexa 2.5mg tablet 1141167976

2. For controls
    - Mood disorders (ICD codes F32, F33, F34, F38, F39 or non-cancer illness code 1286)
    - Treatment/medication codes for antidepressants (Field 20003):
        - allegron 10mg tablet 1140867820
        - amitriptyline hydrochloride+perphenazine 10mg/2mg tablet 1140867948
        - amitriptyline 1140879616
        - amitriptyline+chlordiazepoxide 12.5mg/5mg capsule 1140867938
        - anafranil 10mg capsule 1140867690

- cipralex 5mg tablet 1141190158
- cipramil 10mg tablet 1141151946
- citalopram 1140921600
- clomipramine 1140879620
- cymbalta 30mg gastro-resistant capsule 1141201834
- depixol 3mg tablet 1140867152
- dosulepin 1140909806
- dothiepin 1140879628
- doxepin 1140867640
- duloxetine 1141200564
- edronax 4mg tablet 1141151982
- efexor 37.5mg tablet 1140916288
- escitalopram 1141180212
- faverin 50mg tablet 1140867860
- fluanxol 500micrograms tablet 1140867952
- fluoxetine 1140879540
- flupenthixol 1140867150
- flupentixol 1140909800
- fluphenazine hydrochloride+nortriptyline 1.5mg/30mg tablet 1140867940
- fluvoxamine 1140879544
- imipramine 1140879630
- isocarboxazid 1140867856
- lofepramine 1140867726
- lustral 50mg tablet 1140867884
- manerix 150mg tablet 1140867922
- maoi - tranylcypromine 1140910820
- mianserin 1140879556
- mirtazapine 1141152732
- moclobemide 1140867920
- molipaxin 50mg capsule 1140882244
- nardil 15mg tablet 1140867852

- nortriptyline 1140867818
- oxactin 20mg capsule 1141174756
- parnate 10mg tablet 1140867916
- paroxetine 1140867888
- phenelzine 1140867850
- prothiaden 25mg capsule 1140867624
- prozac 20mg capsule 1140867876
- reboxetine 1141151978
- seroxat 20mg tablet 1140882236
- sertraline 1140867878
- st john's wort/hypericum [ctsu] 1201
- sinequan 10mg capsule 1140882312
- surmontil 10mg tablet 1140867758
- tofranil 10mg tablet 1140867712
- tranylcypromine 1140867914
- tranylcypromine+trifluoperazine 10mg/1mg tablet 1140867944
- trazodone 1140879634
- trimipramine 1140867756
- triptafen tablet 1140867934
- tryptophan product 1140867960
- venlafaxine 1140916282
- yentreve 20mg gastro-resistant capsule 1141200570
- zispin 30mg tablet 1141152736

Table S1. Association between covariates and total brain volume

Variable	Beta ± SE	P
Ethnicity	-5373 ± 1815	**0.003**
Smoking		
Never vs Current	2157 ± 2866	0.452
Never vs Previous	-15294 ± 2926	**1.74E-7**
Alcohol		
Never vs Daily	-21450 ± 2357	**9.781E-20**
Never vs 3-4 times a week	-5108 ± 2218	0.021
Never vs 1-2 times a week	5440 ± 2227	0.015
Never vs 1-3 times a month	12336 ± 2484	**6.893E-7**
Never vs Special occasions	4257 ± 2537	0.093
educational qualification	1922 ± 1032	0.063
Dietary variation	-316 ± 1070	0.767
Dietary change	-6743 ± 1045	**1.142E-10**
BMI	-781 ± 116	**2.48E-11**
Townsend deprivation index	965 ± 192	**5.245E-7**
Physical activity (MET score)	-0.079 ± 0.245	0.747

Table S2. Association of dietary intakes, depression and diet status with global brain volumes

Traits	GM		WM		TBV	
	β±SE	P	β±SE	P	β±SE	P
**Unadjusted**						
Vitamin B6	-6,337.40±953.00	**3.1E-11**	633.45±807.57	0.43	-5,703.93±1,435.89	**7.1E-05**
Vitamin B12	-851.80±171.37	**6.8E-07**	-159.34±144.98	0.27	-1,011.14±257.80	**8.8E-05**
Folate	-48.78±6.33	**1.5E-14**	0.87±5.37	0.87	-47.92±9.54	**5.2E-07**
Depression	8,603.17±1,255.06	**7.8E-12**	-269.30±1,063.81	0.80	8,333.94±1,890.86	**1.06E-05**
Diet status	-7,184.55±5,380.82	0.18	-1,253.70±4,574.93	0.78	-8,438.49±8,092.11	0.29
***Adjusted**						
Vitamin B6	-401.6±852.31	0.64	1207.3±936.46	0.2	805.71±1431.19	0.57
Vitamin B12	-63.58±130.08	0.63	16.97±142.95	0.91	-46.61±218.44	0.83
Folate	-13.57±5.42	**0.01**	5.84±5.96	0.33	-7.73±9.1	0.4
Depression	2,096.40±944.44	**0.03**	267.56±1,038.22	0.80	2,364.03±1,586.25	0.14
Diet status	7,595.36±3,974.37	0.06	1,040.96±4,411.82	0.81	8,636.05±6,689.43	0.20

*Adjusted for age, sex, BMI, ethnicity, smoking, alcohol, Townsend deprivation index, total energy intake, dietary variation, physical activity (MET score) and educational qualification. Values in bold represent statistically significant findings.

Table S3. Association of dietary intakes, depression and diet status with subcortical brain volumes

Traits	Vitamin B6		Vitamin B12		Folate		Depression		Diet status	
	β±SE	P	β±SE	P	β±SE	P	β±SE	P	β±SE	P
**Unadjusted**										
Hippocampus (left)	20.64±9.30	0.02	-1.58±1.67	0.34	0.17±0.06	0.007	-19.74±12.22	0.10	88.04±52.61	0.09
Hippocampus (right)	25.80±9.65	0.008	0.13±1.74	0.93	0.18±0.06	0.005	-15.57±12.71	0.22	64.58±54.08	0.23
Thalamus (left)	64.67±14.81	**1.28E-05**	5.13±2.66	0.05	0.50±0.10	**3.3E-07**	-75.22±19.50	**1.1E-04**	188.12±83.60	0.02
Thalamus (right)	60.75±14.33	**2.28E-05**	4.93±2.57	0.05	0.47±0.10	**9.08E-07**	-74.39±18.87	**8.1E-05**	192.51±81.23	0.01
Caudate (left)	25.40±8.28	**0.002**	2.53±1.49	0.08	0.22±0.05	**7.1E-05**	-40.28±10.90	**2.2E-04**	94.48±47.17	0.04
Caudate (right)	31.63±8.65	**2.5E-04**	2.64±1.56	0.09	0.28±0.06	**1.41E-06**	-38.11±11.40	**0.001**	99.84±49.20	0.04
Putamen (left)	42.52±11.64	**2.6E-04**	3.66±2.09	0.08	0.29±0.08	**1.8E-04**	-58.82±15.32	**1.2E-04**	140.68±65.27	0.03
Putamen (right)	48.00±11.44	**2.7E-04**	5.70±2.05	0.006	0.33±0.08	**1.6E-05**	-61.75±15.06	**4.1E-05**	161.29±64.18	0.01
Pallidum (left)	16.97±4.53	**1.8E-04**	2.30±0.81	0.005	0.13±0.03	**2.3E-05**	-26.00±5.97	**1.3E-05**	57.54±25.82	0.02
Pallidum (right)	13.19±4.56	0.004	2.39±0.82	**0.003**	0.12±0.03	**1.1E-04**	-32.17±5.99	**8.2E-08**	51.14±25.70	0.04
Amygdala (left)	13.48±4.92	0.006	2.51±0.88	0.005	0.15±0.03	**8.9E-06**	-20.57±6.48	**0.002**	30.71±28.00	0.27
Amygdala (right)	20.50±5.46	**1.7E-04**	1.86±0.98	0.058	0.16±0.04	**1.1E-05**	-23.22±7.19	**0.001**	51.57±31.18	0.09
Accumbens (left)	3.90±2.35	0.09	-0.03±0.42	0.95	0.03±0.02	0.089	-1.37±3.09	0.658	15.00±13.09	0.25
Accumbens (right)	3.21±2.21	0.14	0.08±0.40	0.84	0.03±0.01	0.072	0.12±2.91	0.968	6.31±12.37	0.61
***Adjusted**										
Hippocampus (left)	10.9±10.55	0.3	-1.19±1.61	0.46	0.15±0.07	0.02	4.00±11.66	0.73	76.14±49.66	0.13
Hippocampus (right)	-2.36±11	0.83	-0.54±1.68	0.75	0.05±0.07	0.51	11.72±12.16	0.34	46.16±51.15	0.37

Thalamus (left)	0.46±14.88	0.98	3.89±2.27	0.09	0.23±0.09	0.02	-3.45±16.47	0.83	134.66±69.70	0.05
Thalamus (right)	-11.5±14.28	0.42	3.22±2.18	0.14	0.13±0.09	0.15	-1.25±15.81	0.94	141.42±67.42	0.04
Caudate (left)	-9.35±9.47	0.32	0.96±1.45	0.51	0.03±0.06	0.57	-2.70±10.48	0.80	63.48±44.81	0.16
Caudate (right)	-6.54±9.92	0.51	0.58±1.52	0.7	0.07±0.06	0.25	1.99±10.99	0.86	63.72±46.93	0.17
Putamen (left)	-8.89±12.08	0.46	2.8±1.84	0.13	0.02±0.08	0.78	3.79±13.37	0.78	98.79±56.59	0.08
Putamen (right)	-2.5±11.76	0.83	4.44±1.79	0.01	0.07±0.07	0.32	6.27±13.02	0.63	107.67±54.86	0.05
Pallidum (left)	-3.84±5.13	0.45	1.15±0.78	0.14	0.01±0.03	0.71	-2.71±5.69	0.63	34.73±24.25	0.15
Pallidum (right)	-2.37±5.1	0.64	1.79±0.78	0.02	0.03±0.03	0.28	-10.29±5.66	0.07	31.73±23.80	0.18
Amygdala (left)	-1.45±5.75	0.8	1.46±0.88	0.1	0.08±0.04	0.04	-0.48±6.37	0.94	6.06±26.93	0.82
Amygdala (right)	2.68±6.41	0.68	0.63±0.98	0.52	0.07±0.04	0.09	-0.74±7.09	0.92	24.67±30.29	0.42
Accumbens (left)	1.92±2.57	0.46	0.35±0.39	0.37	0.02±0.02	0.18	1.73±2.85	0.54	16.11±11.99	0.18
Accumbens (right)	2.58±2.39	0.28	0.54±0.36	0.14	0.03±0.02	0.05	0.90±2.65	0.73	9.95±11.17	0.37

*Adjusted for age, sex, BMI, ethnicity, smoking, alcohol, Townsend deprivation index, total energy intake, dietary variation, physical activity (MET score), educational qualification and total brain volume. Values in bold represent statistically significant findings.

Table S4. Association of vitamin B6, B12 and folate intake with global brain volumes based on depression status

Dietary variables	Control (n=3926)			Depression (n=2191)			Interaction (n=6117)
	β	SE	P	β	SE	P	P
**Grey matter volume**							
Vitamin B6 intake	930.61	1341.11	0.49	2053.41	1797.22	0.25	0.20
Vitamin B12 intake	20.62	167.99	0.90	-224.31	218.25	0.30	0.66
Folate intake	-16.61	8.32	**0.045**	-21.14	11.34	0.06	**0.02**
**White matter volume**							
Vitamin B6 intake	760.89	1487.73	0.61	1548.73	1947.88	0.43	0.67
Vitamin B12 intake	-7.41	186.35	0.97	-9.04	236.55	0.97	0.95
Folate intake	-0.94	9.23	0.92	7.77	12.29	0.53	0.93
**Total brain volume**							
Vitamin B6 intake	1691.44	2252.20	0.45	3602.25	3024.54	0.23	0.30
Vitamin B12 intake	13.22	282.11	0.96	-233.36	367.30	0.53	0.76
Folate intake	-17.55	13.98	0.21	-13.37	19.09	0.48	0.33

Adjusted for age, sex, BMI, ethnicity, smoking, alcohol, Townsend deprivation index, total energy intake, dietary variation, physical activity (MET score) and educational qualification. Values in bold represent statistically significant findings.

Table S5. Association of vitamin b6, b12 and folate intake with subcortical brain volumes based on depression status

Subcortical volume	Control			Depression			Interaction
	β	SE	P	β	SE	P	P
**Vitamin B6**							
Hippocampus (left)	4.14	16.74	0.80	-10.52	21.88	0.63	0.97
Hippocampus (right)	-4.57	17.63	0.80	-14.17	22.38	0.53	0.80
Thalamus (left)	-19.89	23.17	0.39	-70.83	31.80	0.03	0.07
Thalamus (right)	-23.13	22.23	0.30	-75.25	30.57	0.01	0.04
Caudate (left)	-11.73	15.03	0.44	-39.51	19.65	0.04	0.12
Caudate (right)	-13.38	15.72	0.39	-37.52	20.57	0.07	0.15
Putamen (left)	-15.02	19.08	0.43	-32.85	25.34	0.19	0.31
Putamen (right)	-16.80	18.58	0.37	-30.86	24.58	0.21	0.32
Pallidum (left)	-17.34	8.08	0.03	3.91	10.81	0.72	0.09
Pallidum (right)	-16.35	7.96	0.04	-2.48	10.85	0.82	0.07
Amygdala (left)	-15.96	9.14	0.08	-13.90	11.89	0.24	0.10
Amygdala (right)	-11.09	10.16	0.27	2.83	13.35	0.83	0.53
Accumbens (left)	1.68	4.06	0.68	-4.26	5.40	0.43	0.70
Accumbens (right)	1.79	3.79	0.64	-5.86	4.99	0.24	0.55
**Vitamin B12**							
Hippocampus (left)	1.92	2.09	0.36	-6.78	2.65	0.01	0.04
Hippocampus (right)	1.06	2.21	0.63	-2.48	2.71	0.36	0.65
Thalamus (left)	7.31	2.90	0.01	-0.07	3.86	0.98	0.07
Thalamus (right)	6.43	2.78	0.02	0.34	3.71	0.93	0.12
Caudate (left)	0.50	1.88	0.79	3.03	2.38	0.20	0.39
Caudate (right)	-0.49	1.97	0.81	3.62	2.50	0.15	0.37
Putamen (left)	3.79	2.39	0.11	2.66	3.08	0.39	0.20
Putamen (right)	5.04	2.32	0.03	4.74	2.98	0.11	0.03
Pallidum (left)	2.80	1.01	0.005	-1.01	1.31	0.44	0.01
Pallidum (right)	3.61	1.00	**0.0002**	-0.55	1.32	0.68	**0.001**
Amygdala (left)	2.29	1.14	0.045	0.98	1.44	0.50	0.10
Amygdala (right)	1.12	1.27	0.38	-0.06	1.62	0.97	0.68
Accumbens (left)	0.38	0.51	0.45	0.32	0.66	0.63	0.71
Accumbens (right)	0.93	0.47	0.048	-0.09	0.61	0.88	0.23
**Folate**							

Hippocampus (left)	0.12	0.10	0.23	0.22	0.14	0.11	0.09
Hippocampus (right)	0.04	0.11	0.73	0.14	0.14	0.32	0.54
Thalamus (left)	0.24	0.14	0.09	0.56	0.20	0.005	**0.0032**
Thalamus (right)	0.19	0.14	0.17	0.42	0.19	0.03	0.03
Caudate (left)	0.09	0.09	0.35	0.14	0.12	0.25	0.30
Caudate (right)	0.12	0.10	0.23	0.21	0.13	0.11	0.14
Putamen (left)	0.03	0.12	0.77	0.20	0.16	0.22	0.47
Putamen (right)	0.12	0.12	0.31	0.18	0.16	0.23	0.29
Pallidum (left)	0.06	0.05	0.25	0.01	0.07	0.84	0.38
Pallidum (right)	0.10	0.05	0.048	0.02	0.07	0.78	0.13
Amygdala (left)	0.10	0.06	0.08	0.18	0.07	0.02	0.02
Amygdala (right)	0.11	0.06	0.09	0.06	0.08	0.45	0.17
Accumbens (left)	0.01	0.03	0.70	0.05	0.03	0.17	0.30
Accumbens (right)	0.02	0.02	0.44	0.06	0.03	0.08	0.07

Adjusted for age, sex, ethnicity, BMI, smoking, alcohol, Townsend deprivation index, total energy intake, dietary variation, physical activity (MET score, educational qualification and total brain volume. Values in bold represent statistically significant findings.

Table S6. Association of vitamin b6, b12 and folate intake with global brain volumes after stratifying for depression status and diet groups

**Dietary variables**	**Vegetarian**							**Non-vegetarian**						
	**Control (n=40)**			**Depression (n=38)**			**Interaction (n=78)**	**Control (n=2253)**			**Depression (n=1088)**			**Interaction (n=3341)**
	**β**	**SE**	**P**	**β**	**SE**	**P**	**P**	**β**	**SE**	**P**	**β**	**SE**	**P**	**P**
**Grey matter volume**														
**Vitamin B6 intake**	-23001.42	20573.29	0.28	-2681.77	11629.43	0.82	0.51	192.86	1769.71	0.91	-937.63	2569.33	0.72	0.94
**Vitamin B12 intake**	-8337.20	6003.95	0.18	2642.17	5195.98	0.62	0.44	45.20	222.54	0.84	-22.52	313.13	0.94	0.96
**Folate intake**	68.93	98.39	0.49	227.80	76.10	**0.01**	0.30	-19.54	11.22	0.08	5.74	16.59	0.73	0.29
**White matter volume**														
**Vitamin B6 intake**	27640.29	17057.07	0.12	-23108.10	15270.78	0.15	0.07	-1609.96	2020.29	0.43	1526.95	2699.46	0.57	0.79
**Vitamin B12 intake**	-6201.06	4977.81	0.23	-11237.86	6822.92	0.12	0.09	229.44	254.05	0.37	-332.58	328.99	0.31	0.26
**Folate intake**	-119.82	81.57	0.16	160.64	99.93	0.12	**0.02**	3.96	12.81	0.76	14.03	17.43	0.42	0.87
**Total brain volume**														
**Vitamin B6 intake**	4637.61	29443.24	0.88	-25792.24	21931.60	0.25	0.12	-1417.05	3019.10	0.64	589.70	4224.30	0.89	0.96
**Vitamin B12 intake**	-14538.70	8592.49	0.11	-8596.70	9798.95	0.39	0.12	274.68	379.65	0.47	-355.12	514.82	0.49	0.46
**Folate intake**	-50.88	140.81	0.72	388.45	143.52	**0.01**	0.09	-15.58	19.14	0.42	19.76	27.28	0.47	0.78

Adjusted for age, sex, ethnicity, BMI, smoking, alcohol, Townsend deprivation index, total energy intake, dietary variation, physical activity (MET score), and educational qualification. Values in bold represent statistically significant findings.

Table S7. Association of vitamin b6, b12 and folate intake with subcortical brain volumes after stratifying for depression status and diet groups

Subcortical volume	Vegetarian							Non-vegetarian						
	Control			Depression			Interaction	Control			Depression			Interaction
	β	SE	P	β	SE	P	P	β	SE	P	β	SE	P	P
**Vitamin B6**														
Hippocampus (left)	-90.48	234.56	0.70	191.60	319.85	0.56	0.99	-43.24	31.77	0.17	-27.88	22.29	0.21	0.17
Hippocampus (right)	10.97	218.43	0.96	264.56	287.09	0.37	0.77	-60.85	31.11	0.05	-34.25	23.25	0.14	0.07
Thalamus (left)	-89.78	304.58	0.77	-183.25	327.74	0.58	0.53	-103.80	45.46	0.02	-46.91	30.96	0.13	0.03
Thalamus (right)	-150.27	285.53	0.61	-495.06	288.07	0.10	0.12	-106.55	43.95	0.02	-52.59	29.92	0.08	0.01
Caudate (left)	-51.78	174.21	0.77	-301.81	229.60	0.20	0.33	-49.25	28.27	0.08	-25.32	20.29	0.21	0.13
Caudate (right)	-273.68	149.20	0.08	-201.86	252.70	0.43	0.14	-37.38	29.59	0.21	-37.05	21.24	0.08	0.11
Putamen (left)	-27.32	323.38	0.93	-318.59	259.49	0.23	0.76	-51.82	36.21	0.15	-53.49	25.43	0.04	0.04
Putamen (right)	-193.00	236.46	0.43	-443.79	249.18	0.09	0.21	-37.45	35.14	0.29	-51.97	24.67	0.04	0.07
Pallidum (left)	-55.28	73.98	0.46	-5.38	126.28	0.97	0.82	5.18	15.62	0.74	-28.85	10.87	0.008	0.07
Pallidum (right)	-115.20	85.14	0.19	-109.88	104.39	0.31	0.14	0.36	15.84	0.98	-22.29	10.45	0.03	0.14
Amygdala (left)	-124.54	120.96	0.32	57.04	125.83	0.66	0.59	2.18	17.09	0.90	-17.54	12.06	0.15	0.51
Amygdala (right)	-69.33	132.17	0.61	231.76	159.53	0.16	0.53	9.90	19.65	0.61	-8.24	13.47	0.54	0.85
Accumbens (left)	-8.72	41.43	0.84	14.44	51.41	0.78	0.86	-0.85	7.77	0.91	1.67	5.37	0.76	0.97
Accumbens (right)	-22.82	39.07	0.57	-102.16	54.04	0.07	0.04	-3.82	7.23	0.60	3.61	4.99	0.47	0.52
**Vitamin B12**														
Hippocampus (left)	185.64	99.44	0.08	57.16	103.21	0.59	0.47	2.10	2.80	0.45	-7.19	3.87	0.06	0.17
Hippocampus (right)	106.11	89.26	0.25	-100.50	96.99	0.31	0.51	-1.88	2.93	0.52	-4.23	3.79	0.27	0.44
Thalamus (left)	99.30	101.90	0.34	-34.19	134.02	0.80	0.69	3.25	3.89	0.40	-2.65	5.55	0.63	0.64
Thalamus (right)	159.86	89.56	0.09	-49.75	125.64	0.70	0.46	4.07	3.76	0.28	-1.19	5.37	0.82	0.62

Caudate (left)	49.70	71.38	0.49	57.95	76.66	0.46	0.51	-1.42	2.56	0.58	-1.26	3.45	0.71	0.75
Caudate (right)	59.19	78.57	0.46	-0.29	65.65	1.00	0.62	-1.19	2.68	0.66	-0.98	3.61	0.79	0.83
Putamen (left)	81.43	80.68	0.32	-56.15	142.29	0.70	0.17	0.65	3.20	0.84	3.60	4.42	0.42	0.84
Putamen (right)	112.58	77.47	0.16	-136.66	104.05	0.21	0.07	1.00	3.09	0.75	2.46	4.29	0.57	0.88
Pallidum (left)	41.34	39.26	0.30	-55.14	32.55	0.11	0.17	0.84	1.37	0.54	-0.64	1.91	0.74	0.46
Pallidum (right)	13.97	32.45	0.67	-23.70	37.47	0.54	0.52	1.94	1.31	0.14	-0.99	1.94	0.61	0.22
Amygdala (left)	-1.27	39.12	0.97	24.29	53.23	0.65	0.94	0.95	1.51	0.53	1.71	2.08	0.41	0.71
Amygdala (right)	12.93	49.60	0.80	99.14	58.16	0.11	0.36	-1.34	1.69	0.43	-1.41	2.41	0.56	0.57
Accumbens (left)	24.26	15.98	0.14	18.94	18.23	0.31	0.50	0.63	0.67	0.35	0.46	0.95	0.63	0.62
Accumbens (right)	4.16	16.80	0.81	-6.98	17.19	0.69	0.90	0.54	0.63	0.39	-0.12	0.88	0.89	0.79
**Folate**														
Hippocampus (left)	-0.02	1.53	0.99	0.23	1.74	0.90	0.76	0.26	0.14	0.06	0.32	0.21	0.12	0.03
Hippocampus (right)	-0.28	1.38	0.84	-1.74	1.63	0.30	0.93	0.24	0.15	0.10	0.30	0.20	0.14	0.08
Thalamus (left)	0.77	1.57	0.63	-1.50	2.27	0.52	0.93	0.39	0.20	0.05	0.58	0.29	0.046	0.01
Thalamus (right)	1.67	1.38	0.24	0.15	2.12	0.94	0.55	0.31	0.19	0.11	0.51	0.28	0.07	0.04
Caudate (left)	0.02	1.10	0.98	0.86	1.30	0.51	0.58	0.16	0.13	0.22	0.12	0.18	0.49	0.38
Caudate (right)	-0.42	1.21	0.73	1.78	1.11	0.13	0.32	0.20	0.13	0.15	0.11	0.19	0.55	0.29
Putamen (left)	-0.77	1.24	0.55	1.63	2.40	0.51	0.82	0.12	0.16	0.47	0.22	0.23	0.35	0.54
Putamen (right)	0.38	1.19	0.75	-0.03	1.76	0.98	0.69	0.14	0.16	0.37	0.18	0.23	0.44	0.48
Pallidum (left)	0.10	0.61	0.86	0.61	0.55	0.28	0.41	0.12	0.07	0.09	0.03	0.10	0.75	0.12
Pallidum (right)	1.20	0.50	0.03	0.88	0.63	0.18	0.02	0.12	0.07	0.07	0.01	0.10	0.92	0.19
Amygdala (left)	0.32	0.60	0.60	1.09	0.90	0.24	0.35	0.15	0.08	0.06	0.15	0.11	0.17	0.05
Amygdala (right)	-0.52	0.76	0.50	1.45	0.98	0.16	0.74	0.11	0.09	0.18	0.06	0.13	0.63	0.32
Accumbens (left)	-0.22	0.25	0.39	-0.08	0.31	0.79	0.84	-0.02	0.03	0.60	0.01	0.05	0.88	0.86
Accumbens (right)	0.33	0.26	0.21	0.10	0.29	0.73	0.047	0.001	0.03	0.97	0.06	0.05	0.23	0.30

Adjusted for age, sex, ethnicity, BMI, smoking, alcohol, Townsend deprivation index, total energy intake, dietary variation, physical activity (MET score), educational qualification and total brain volume. Values in bold represent statistically significant findings.
